# Investor demand in syndicated EFSF/ESM bond issuances

**DOI:** 10.12688/openreseurope.15961.2

**Published:** 2024-07-16

**Authors:** Martin Hillebrand, Marko Mravlak, Peter Schwendner

**Affiliations:** 1 XU Exponential University of Applied Sciences, Potsdam, Brandenburg, Germany; 2 European Stability Mechanism, Luxembourg, 1347, Luxembourg; 3 ZHAW Zurcher Hochschule fur Angewandte Wissenschaften, Winterthur, Zurich, 8401, Switzerland

**Keywords:** fixed income, European sovereign debt crisis, primary bond markets, orderbooks

## Abstract

The European Financial Stability Facility (EFSF) and the European Stability Mechanism (ESM) were set up at the peak of the European sovereign debt crisis to issue bonds and lend to countries under current funding stress. This study analyses investor demand in syndicated bond issuances of EFSF and ESM from 2014 to 2020 on an unprecedented granularity level using a dataset of individual orders with statistical inference. Particularly, we investigate orderbook dynamics for three main aspects: first, we determine the main factors segmenting investor demand. Second, we analyse price dynamics in the transactions and their relation to investor demand. Third, we investigate whether any indications of orderbook inflation might explain the increased volatility in orderbook volume.

We identify issuance tranche and tenor as the main determinants of investor demand that are largely anticipated in the notional. Further, we note that ESM is doing economical pricing, where the new issue premium tends to be lower in a market context with larger demand. Lastly, we find a mixture of an increasing number and an increasing volume of orders as drivers of large order books. This confirms that there are no indications of orderbook inflation tendencies in the analysed time period.

## Plain language summary

During the European debt crises, specific Euro area countries lost their access to the private capital markets to cover their budget deficits. For this reason, all Euro area countries set up the European Financial Stability Facility (EFSF) and the European Stability Mechanism (ESM) to jointly collect funding on the private capital markets via bonds and offer loans to those countries that needed financial assistance. The preferences and behaviour of investors who bought these bonds on the primary market (i.e. directly from the issuer) can be statistically analysed. The results show investors mainly look at the time-to-maturity of the bonds and if those bonds already exist on the secondary market (and could be bought from another investor). On the primary market, bonds are offered a bit cheaper than the secondary market price (“new issue premium”) to ensure there is always enough demand. A second finding is that the new issue premium tends to be lower in situations where the demand is high. Thirdly, there is no sign of significant “orderbook inflation”, so investors don't seem to tactically inflate the size of their orders beyond their actual demand to get a higher allocation.

## Introduction

Solid primary market investor demand is a fundamental prerequisite of the ability of a supranational institution to cover its financing needs directly in the capital market. Understanding the investor demand dynamics is of outmost importance for the issuer to raise funds cost-effectively and access the market successfully even in turbulent market conditions: the more closely the tenor, issuance volume, and issuance format align with investors' preferences, the fewer financial concessions are required to attract a sufficient number of buyers for the issued bonds. This paper looks at three crucial aspects of investor demand: first, it looks at the specific investor demand situation for different market segments in terms of tranche and tenor. Second, it analyses how investor demand is connected to price dynamics, particularly the new issuance premium (NIP) that the issuer can offer to attract investors. Third, it investigates drivers of extreme demand dynamics, namely large orderbooks. This analysis is able to give new insights into investor specific behaviour because it is based on a dataset of unique size and granularity, containing more than 10,000 orders of about 100 syndicated bond transactions and more than 14,000 investors which are categorised by investor type. In contrast to auctioned transactions, syndicated transactions usually have a gradual price-building allowing investors to react with adjusted orders.

## Literature review and derivation of hypotheses

Syndicate banks, also called (co-)lead managers or underwriters, are usually obliged to buy the uncovered amounts of the bond issuance they could not place at their investor base. This risk is covered by the issuance fee paid from the issuer to the syndicate banks. Therefore, they are keen to fill the orderbook quickly and have a certain oversubscription such that the issuer has some freedom when allocating the bonds, in favour of investors that have e.g. a positive effect on the secondary market. Usually, the price discovery during a syndicated transaction leads to a higher price than that in the secondary market, hence showing a positive new issue premium (NIP). Buis *et al.* (2020) highlight the institutional setting of the European primary and secondary sovereign bond markets.

Once a bond is issued, it can be re-opened (tapped). This means that the issuers offer another tranche of the same bond. Economically, it is different whether a new bond is issued or an existing bond is tapped. While in the latter case, a secondary market already exists for this bond, this is not the case for each bond's first (or "inaugural") issuance. Typically, the first issuance has a substantial issuance volume, also called "benchmark size" to enable the development of a sufficiently liquid secondary market. Hence, we subsequently use the term "benchmark issuance" for first issuances. It appears natural to consider the tranche when looking at investor demand of different issuances. The use of taps (reopenings) of bonds has been analysed in detail for auctioned sovereign bonds, e.g. for Italian bonds ( Scalia, 1998), or corporate bonds ( Maul & Schiereck, 2018).

One main characteristic of a bond transaction is the tenor, which is the time to maturity at issuance. The tenor is one of the most important determinants of investor demand. Bonds with long tenors offer usually higher coupons at higher market risk, and many investors are entirely focused on specific tenors. Also, issuers have certain tenor preferences as it is widely discussed in the literature such as recently in Beetsma *et al.* (2021). Many authors suggest methods to optimise the choice of tenor in public debt management. For example, Greenwood *et al.* (2015) and Zenios *et al.* (2021) suggest an optimal choice of tenors considering both borrowing costs and refinancing risk. In contrast, our focus here is the investor demand aspect of the tenor. As a longer time to maturity implies a higher exposure to market risk, we expect a larger demand for shorter maturities.

Another relevant constituent of a bond transaction is the pricing. Here we focus on the yield difference of the issuance to a comparable yield, most of the time a corresponding secondary market yield, or a composition (e.g. linear combination) of several secondary market bond yields. Usually, the yield difference (also called new issue premium, or NIP) is positive, which means that the issuer is ensuring an attractive yield for the investors to obtain a good liquidity for the newly issued bond. Maitra *et al.* (2018), Maul and Schiereck (2018) and Krebbers *et al.* (2022) analyse new issue premia at the European corporate bond market, whereas the bulk of the literature like Goldberg and Ronn (2013), Ben Dor and Xu (2015), Nagler and Ottonello (2022) and Günster *et al.* (2023) addresses the US corporate bond market. Most studies find positive new issue premia in corporate bond markets. However, Matsui (2006) finds a negative new issue premium for Japanese corporate bond issued from 1995–2000. The issuer is interested to keep the NIP as tight as possible: In market environments that indicate a low investor demand, the NIP needs to be higher to incentivize investors to participate in the transaction, whereas this is not necessary in market environments that indicate large investor demand: there, we expect the NIP to be low. Of course, there are other success factors to be considered as well, yet careful pricing can be regarded as a quality indicator for a transaction. Hence our assumption is that, for ESM bond issuances, investor demand and NIP are negatively correlated. A third phenomenon in investor demand dynamics that we investigate are very large orderbooks. The background to it is as follows: investors expecting to be only partially allocated might increase their order such that the allocated volume they expect would better match their demand. When there are indications of a large demand (e.g. a quick tightening of the price guidance), this might lead to very large order volumes: investors would expect large orders of other investors and therefore increase their order a second time. This means that order volumes would not necessarily show real demand, instead would be an overshooting, by different magnitudes. Indeed, such investor behaviour has been observed in the markets: Ding *et al.* (2022) report significant overpricing for the Chinese corporate bond market measured by weak performance on the first trading day on the secondary market. However, for our dataset of EFSF and ESM bond issuances, we expect that increasing real demand – more investor orders – is causing large orderbooks rather than tactical bidding, seen in unusually larger orders.

The following analysis is based on the primary bond markets of ESM and EFSF. Due to the relevance of ESM and EFSF for financial stability in the Euro area and their key role in supporting the integrity of the Euro area during the European sovereign debt crisis of 2010–2012 ( Boros & Sztanó, 2021; Moessner, 2018) and preventing a further core-periphery divergence, a deeper understanding of their bond markets is of broader interest. Furthermore, since they have jointly been among the largest supranational bond issuers in Euro recently, they can be regarded as important representatives of the European SSA ^1^ segment. In contrast to the cited literature above, we analyse orderbooks on the granular level of individual orders which allows particularly for a distinction between investor types.

This article is structured as follows: First, we look at the *main factors segmenting investor demand*, the tenor (time to maturity at issuance) and the issuance tranche of the bond. The section *Tuning the price: The New Issue Premium* (NIP) discusses the pricing dynamics of the ESM/EFSF bond transactions concerning investor demand and particularly investigate whether there are indications that the NIP is economically used as primary market pick-up for investors, i.e., that the NIP tends to be lower for larger orderbooks and vice versa. The section *Are there indications of Orderbook Inflation* analyses the drivers leading to exceptionally large orderbooks in ESM and EFSF transactions. Specifically, we investigate whether the number of orders is a main explanatory factor for investor demand. We further analyse whether there are indications that investors increase their orders in expectation of large orderbooks, leading to a phenomenon often called "orderbook inflation".

## Methods

### Study design

Our study carries out statistical inference and discusses descriptive statistics of various aggregation levels of investor demand and their correlations. Particularly, the significance of main features is shown using inferential statistics: while the hypotheses on demand patterns with respect to tenor and benchmark or tap issuance are proven with two-sample t-tests, the negative correlation of the NIP with investor demand is shown using bootstrapping. The relationship of order size and number of orders is shown using linear regression, where a t-test proves a significant positive relation. Further patterns are shown with descriptive statistics, supported by suitable graphs. Our goal is to offer public transparency into the aggregate demand of EFSF/ESM bonds on the primary markets and its determinants. We would like to encourage other public bond issuers to provide comparable transparency to foster trust in public funding.

### Data

Our analysis is based on a proprietary data warehouse that holds all orderbooks of ESM and EFSF syndicated transactions from January 2014 to September 2020. Each orderbook consists of the order volumes that investors placed in the particular transaction. Hence these volumes are the natural measure of investor demand, and the last section gives indications that there is no significant bias between placed orders and real demand.

A particular strength of the data set is that investors that appear in different orderbooks are matched, meaning that all orders of the same investor are assigned to this investor. This way it is possible to trace individual investor behaviour over a period of several years. This feature is necessary to analyse the appearance of orderbook inflation as carried out in the last section. Please note that the orderbooks are created by the bookrunner of the respective transaction, one of the syndicate members. Hence the orderbooks are stemming from different sources, and creating a joint data set with matched investors is a non-trivial exercise.

Also, the investors are carefully classified with respect to six different investor types which allows for the analysis of investor type-specific behaviour. Numerous issuance-related characteristics such as the tenor, issuance volume or tranche are included to serve as possible explanatory variables in the analyses. We group the investors into six categories: Central Banks and other Public Institutions (Supranationals, Agencies, Sovereign Health Funds, Municipalities), Banks (inclusive Bank Treasury), Bank Trading, Asset Managers, Brokers / Hedge Funds and Pension Funds / Insurances.

**Figure 1.**
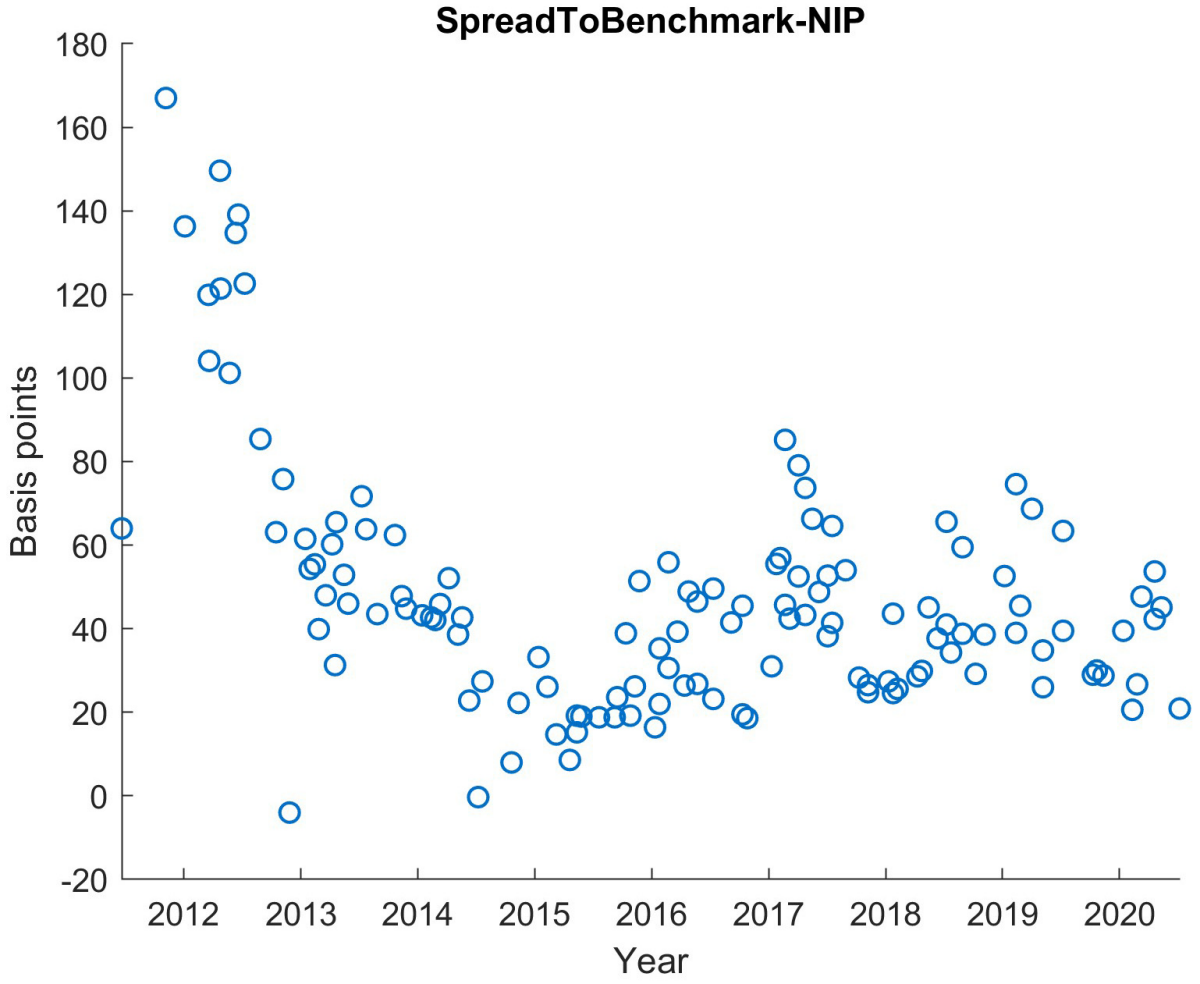
Benchmark Spread in basis points of the bond yield at issuance with NIP subtracted for EFSF and ESM bonds. EFSF & ESM bonds primary market.

For the statistical analyses of this article, we used MATLAB, but a free statistical software like R could also have been used. We exclude all orderbooks before 2014. The reason is the irregular demand structure in the early phase of ESM and EFSF compared to the period since 2014 as seen in Figure 1. It shows the yield advantage of the ESM and EFSF bonds in the secondary market in relation to comparable German bonds (so-called "benchmark bonds" ^2^ in the Euro area). Technically, it is the yield difference (yield spread) of the ESM/EFSF bond at issuance minus its “new issue premium” compared to the yield of the German benchmark bond. From the first issues until the end of 2013, this difference substantially declined, reflecting the successful establishment phase of the ESM as an issuer. Overall, the data set for this analysis, from January 2014 until September 2020, contains 97 syndicated issuances with 11,935 individual orders, a total issuance volume of 259bn EUR and a total order volume of 545bn EUR.

Creating a comparable data set for one or several issuers requires a diligently processing of each orderbook in the observation period close to its issuance data, since the investor matching, even when supported by matching algorithms, requires manual adjustments to achieve a satisfying data quality. The data set used in this analysis has been carefully created over a span of seven years.

## Main market segmenting: benchmarks and taps, maturity buckets

In this section we take a closer look at the characteristics of the notional, the tenor and the issuance tranche (benchmark/tap) as fundamental determinants of investor demand.

## Benchmark versus tap

Generally, tapping a bond decreases the heterogeneity of all outstanding bonds of an issuer. With a tap, the outstanding volume of a bond is increased with usually increases the secondary market liquidity of this instrument. On the other hand, many investors have already satisfied their demand for this bond in the benchmark issuance, leading to the assumption that the demand for taps is usually lower than for benchmarks. In our data sample, not every bond has been tapped, and only very few ones have been tapped more than once. As the main difference between benchmark and tap is the existence of a secondary market for tapped bonds, we do not distinguish between the first and the few second taps of the bonds. Hence, we regard such two taps as two different observations in our sample.

**Figure 2.**
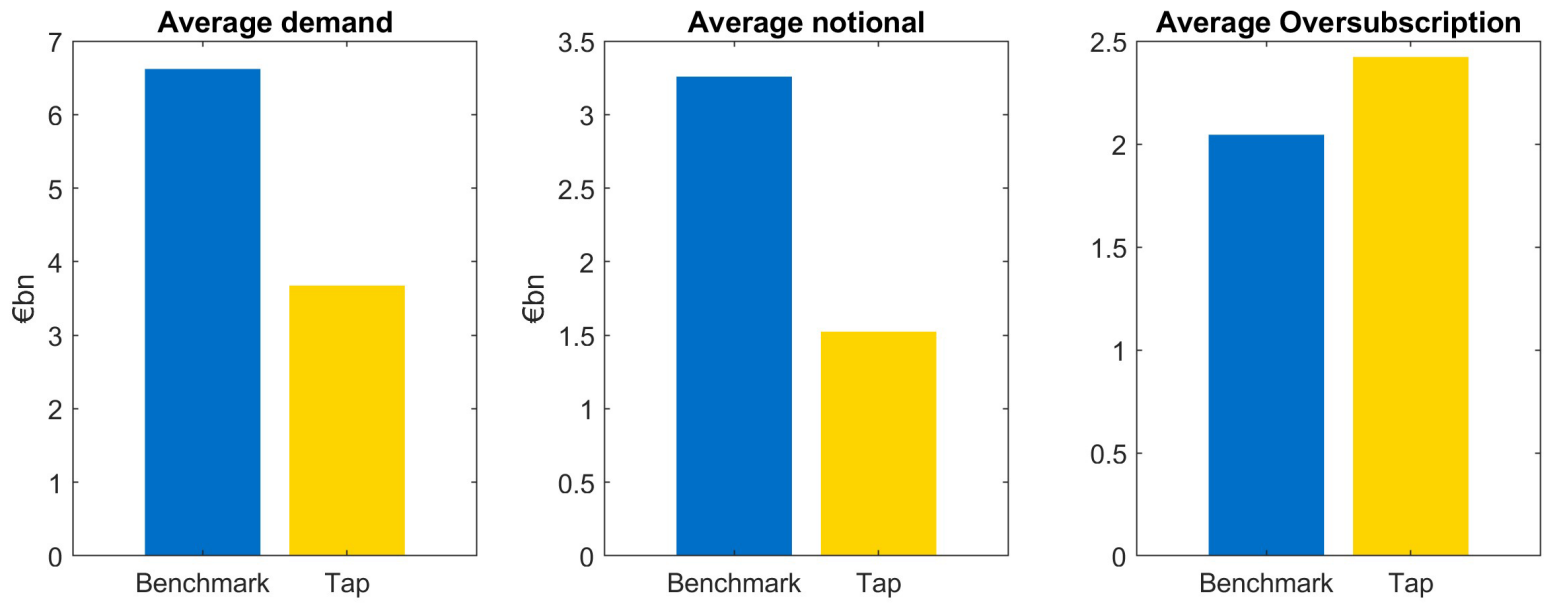
Comparison of average demand, notional and oversubscription for benchmark and tap issuances of EFSF and ESM bonds. EFSF & ESM bonds primary market.

We observe the following characteristics when comparing benchmark issuances and taps: first of all, the average investor demand in benchmark transactions is approximately twice as large as demand of tap issuances, see Figure 2. This relation is highly significant, we can reject the null hypothesis that demand in taps is larger than in benchmark issuances.

The fact that investor demand is substantially lower for taps is already taken into account when deciding the notional of the transaction. Therefore, the difference between benchmark and tap demand is mainly reflected in the different notional. That said, the average notional of taps relative to the demand is smaller than the one of benchmark issuances resulting in a larger average oversubscription of taps as can be seen in Figure 2.

## Tenor buckets

Looking at average investor demand in different tenors, there is a general pattern that long maturities have less demand. However, in detail, the relation is a bit more complex, as it is shown in the subsequent Figures. For a statistical assessment, we group the issuances in four tenor buckets: Short maturities (<3Yr), the 5-year bucket (3Yr–7yr), the 10yr bucket (7Yr-12Yr) and the long maturities (>12Yr). This grouping is consistent to the typical tenor bucket segmentation of the bond market that is even reflected in the definition of bond futures contracts of the most important contracts on the US treasury notes and bonds traded on the CME ^3^ (2Y “ZT” contract, 5Y “ZF” contract, 10Y “ZN” contract, 30Y “ZB” contract) and the German futures contracts (2Y “FGBS” contract, 5Y “FGBM” contract, 10Y “FGBL” contract and 30Y “FGBX” contract) traded on the Eurex ^4^ exchange. There are also Eurex-traded contracts on the sovereign bonds of other European countries (Italy, France, Spain, Switzerland), but not for the full range of tenors.

**Figure 3.**
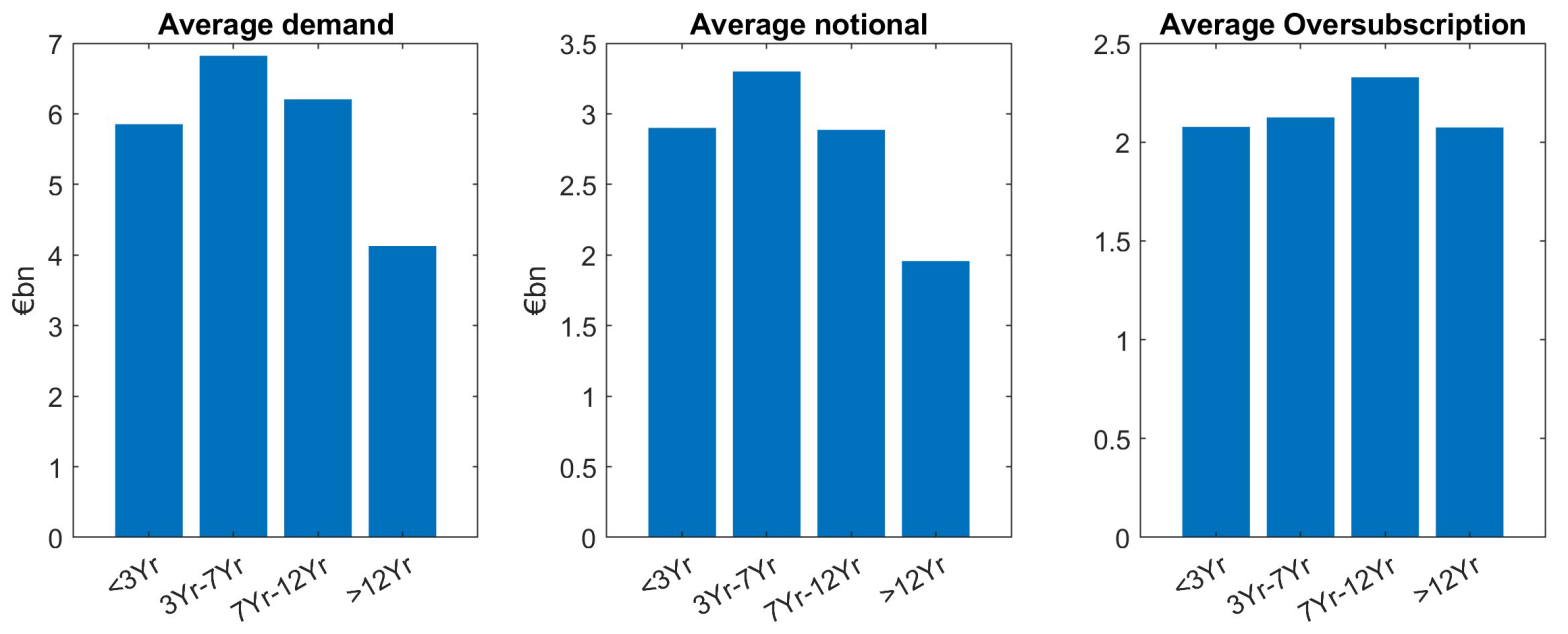
Comparison of average demand, notional and oversubscription for all issuances of EFSF and ESM bonds. EFSF & ESM bonds primary market.

Our main finding is that demand is larger for smaller tenors, except for the short maturities (<3Yr) which is the tenor bucket with the fewest issuances (see Table 1), see the left-hand side panel of Figure 3.

**Table 1.** Significance tests for investor demand in different subgroups: Benchmark/Tap.

	Number of benchmarks	Number of taps	Mean orderbook size benchmarks	Mean orderbook size taps	p-value
All transactions	64	33	6.62	3.67	<0.01%
<3Yr	3	2	7.59	3.25	No statistics
3–7Yr	20	5	7.58	3.79	<0.1%
7–12Yr	23	10	6.95	4.50	2.0%
>12Yr	18	16	4.97	3.18	4.4%

H_0: demand in taps is larger than in benchmark issuances. Result: (number of benchmarks, number of taps, p-value)

The same “lower demand for larger tenors” pattern can be seen for the average notional (mid panel of Figure 3). As oversubscription is defined as the ratio of demand divided by notional (issue size), the trends for demand and notional roughly cancel out, leads to almost the same average oversubscription (right hand side panel) for the different tenor buckets. Grouping issuances in short tenors (<7Yr) and large tenors (≥7Yr), a two-sample *t*-test supports the hypothesis of larger demand for smaller tenors at a 5% significance level (see Table 1– Table 2). This further grouping was necessary as the number of short tenors (<3Yr) was too small to be evaluated separately.

**Table 2.** Significance tests for investor demand in different subgroups: Maturities. EFSF & ESM bonds primary market.

	Number of issuances <7Yr	Number of issuances >7Yr	Mean orderbook size <7Yr	Mean orderbook size ≥7Yr	p-value
All transactions	30	67	6.66	5.15	1.3%
Benchmarks	23	41	7.58	6.08	3.6%
Taps	7	26	3.64	3.68	47.8%

Group Maturities: <7Yr vs ≥7Yr H_0: demand larger in larger issuances than in smaller issuances.

**Figure 4.**
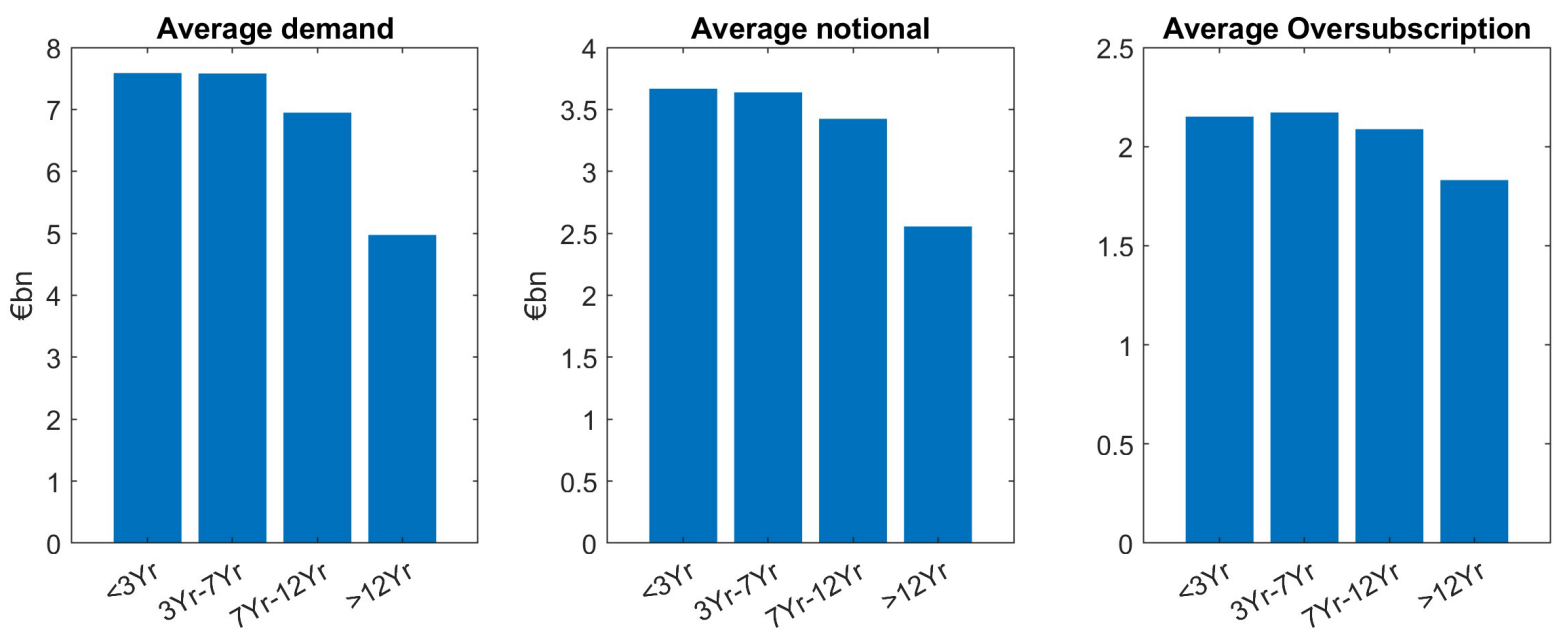
Comparison of average demand, notional and oversubscription for benchmark issuances of EFSF and ESM bonds. EFSF & ESM bonds primary market.

**Figure 5.**
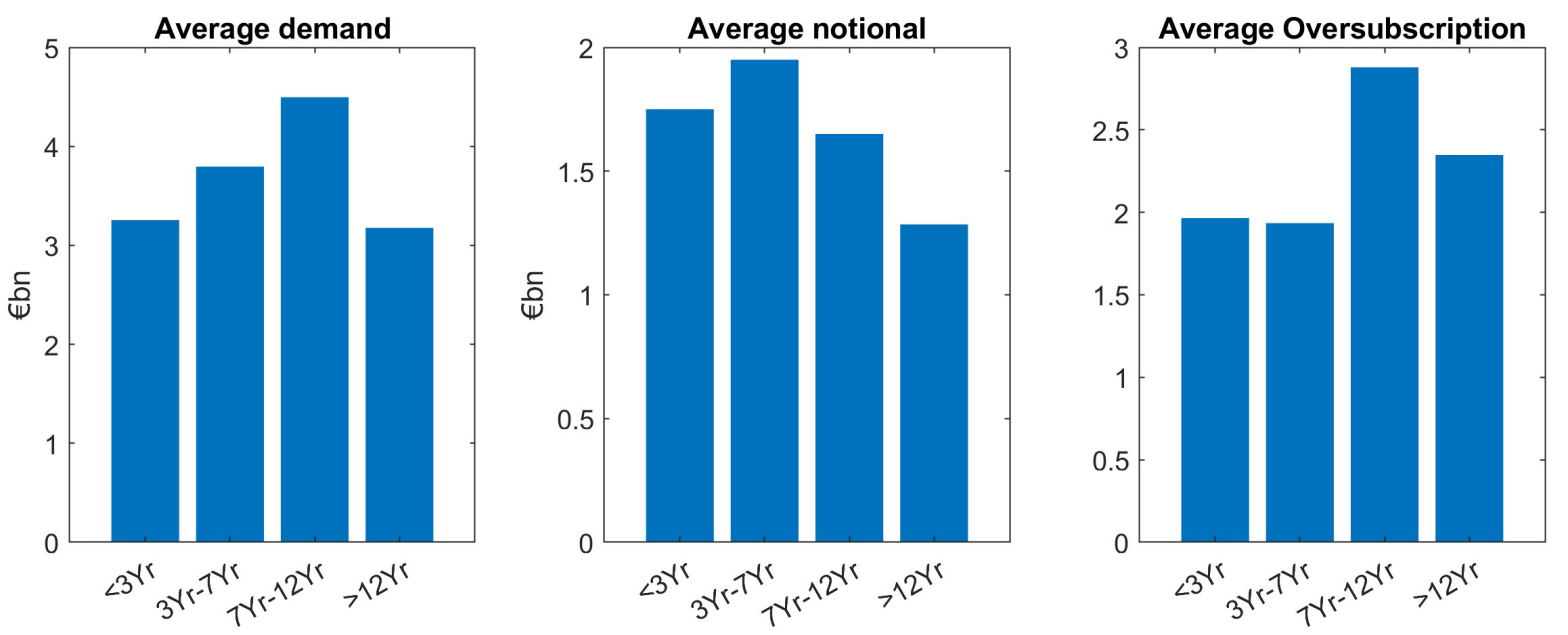
Comparison of average demand, notional and oversubscription for tap issuances of EFSF and ESM bonds. EFSF & ESM bonds primary market.

Looking at benchmarks and taps separately, we confirm our main finding which is larger demand for smaller tenors particularly holds for benchmarks and is significant at a 5% level in a two-sample setup as described above. In contrast to this, tap issuances show (non-significant) higher demand for larger tenors, see Figure 4 and Figure 5. Moreover, for benchmarks, demand for larger tenors is well reflected through smaller notional for larger demand. Also, as we can see in Figure 4, it leads to an overall balanced and only slightly decreasing oversubscription for increasing tenors.

In contrast, for taps, demand for larger tenors is increasing (except for the largest tenor bucket), resulting in higher oversubscriptions for larger tapped tenors, as can be seen in Figure 5.

Overall, across benchmark and tap issuances, we see an almost balanced oversubscription for larger tenors. This indicates that in the average, the different investor demand in the different maturity buckets is very well reflected in the notional. In other words, market demand for different tenor buckets was overall well matched by the corresponding ESM/EFSF supply, see again Figure 3.

## Tuning the price: the new issue premium

The objective of a bond issuance transaction is not only to cover the liquidity needs according to the funding plan in an asset liability management (ALM) sense, but also to support the long-term funding strategy. This long-term strategy includes developing a diversified and reliable primary market investor base and a liquid secondary market. Although ESM is not actively trading in the secondary market, it is a stakeholder, as stable pricing, low volatility and high liquidity contribute to the confidence of primary market investors and lowers the risk premium of ESM issuances relative to the Bund benchmarks. To fulfil these secondary objectives, a certain oversubscription of the orderbook is a necessary condition for the funding and investor relation team to allocate orders to achieve a diversification in terms of investor countries and investor types. As the goal of ESM is to ensure primary market access even in severe stress situations such as during the European sovereign debt crises, a diversified international investor base also outside of Europe is a substantial strategic asset. A moderately oversubscribed orderbook also signals such a solid market access and is hence desired.

At a fixed funding size, maturity schedule and issuance calendar, the new issue premium (NIP) is an adjustment tool set by the funding team to enable their desired allocation. The new issue premium is defined as the yield spread between the new issue and a comparable issue already trading at the secondary market. Naturally, investors can be expected to increase their demand with increased NIP offered by the issuer. Hence, at a too low new issue premium, it might not be possible to implement the desired allocation structure due to a lack of demand and the issued bond might underperform at the first day of trading. Also, a too high new issue premium is undesired. The obvious reason is that it would be an overspending of the programme countries taxpayers funds to investors. It also might motivate additional investors to submit inflated orders and then to immediately sell the new issue on the first trading day and thereby realising the NIP, hence sending an economically wrong signal of risk and underpricing to the market.

If the NIP is set correctly, it supports a modestly positive performance of the new issue at the first trading day, thus sending a stabilising signal to the market. Large sell volumes on the first day on the secondary market or an overly inflated orderbook could be signs of a too generous NIP. As the funding officers set the NIP at the beginning of the book building and possibly adjust it during the book building process, the NIP incorporates not only their expectation, but also their reaction to the received investor demand.

To summarise these considerations, the investor are expected to have *ceteris paribus* larger demand for issuances with larger NIP which would, ceteris paribus, result in a positive correlation between NIP and investor demand. On the other hand, as the NIP is an active tool of the funding officers to react on low demand, the NIP could also be smaller for large orderbooks and higher for smaller orderbooks. In other words: NIP and order volume could be negatively correlated.

Our hypothesis is that the latter effect is the dominating one, leading overall to a negative correlation. As both the NIP set by the issuer and total investor demand already include reciprocal expectations, we put the hypothesis as a correlation, not as a linear relationship between an explained and an explaining variable. Then we discuss the significance of the reported correlation using a bootstrap analysis.

Please note that this analysis focuses on first issuances (benchmarks) only. The reason is that for taps, a secondary market is already existing for exactly this bond, and the danger to cannibalise the secondary market limits the magnitude of the NIP.

**Figure 6.**
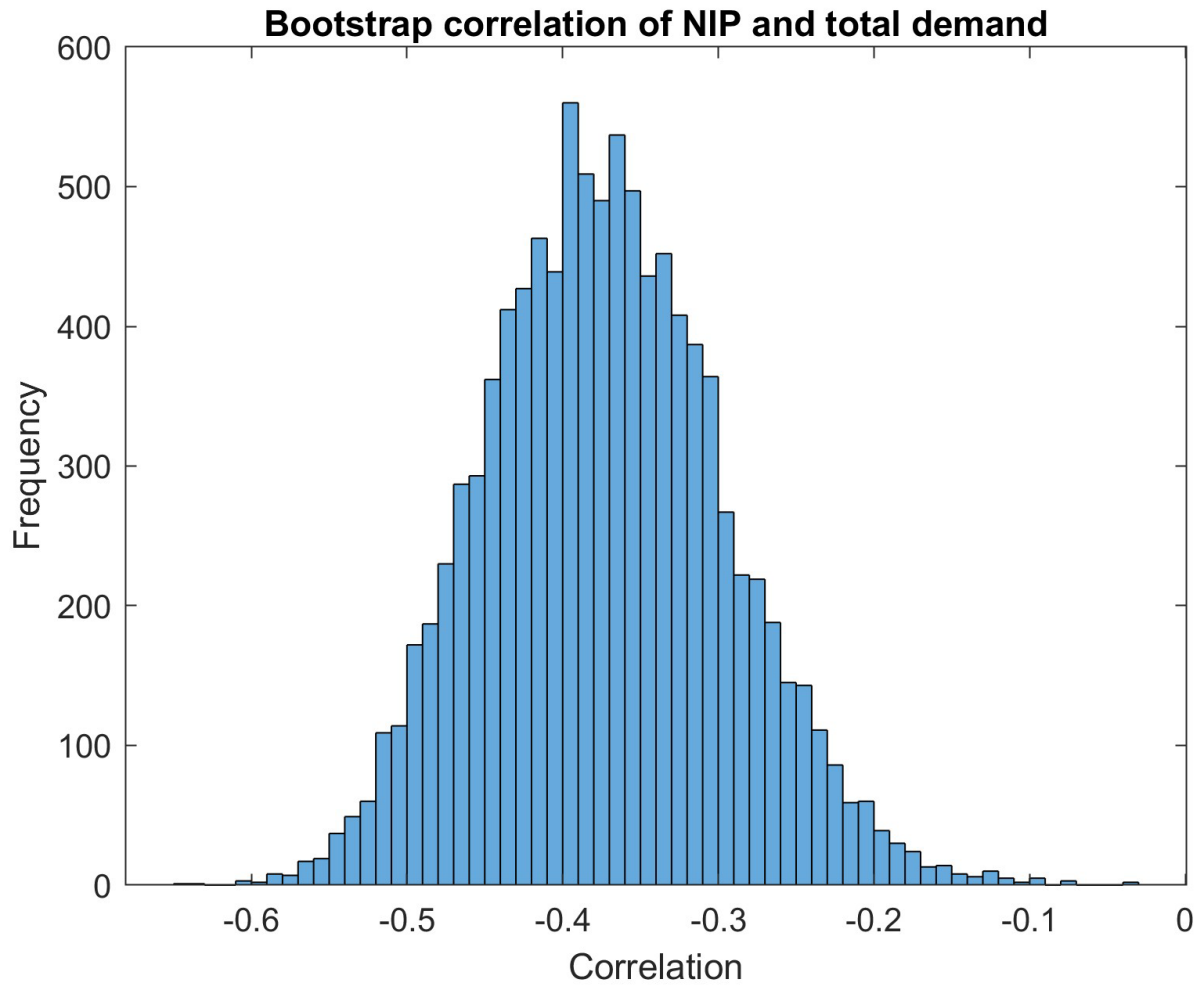
Distribution of correlation of NIP and total demand as calculated by bootstrapping method. EFSF & ESM bonds primary market.

Indeed, we observe a negative correlation of -0.3734 between total demand and NIP in the dataset of all orders between 2014 and September 2020. To assess the statistical validity of the reported correlations, we compute the standard deviation of 10000 bootstrapped correlations. As an example, Figure 6 shows the histogram of 10000 bootstrapped correlations between the total demand and the new issue premium (NIP). The mean of the bootstrap samples is μ=-0.3735. It shows a slight deviation to the empirical correlation due to the random sampling. The standard deviation of the bootstrap samples is σ=0.0761. We are using the standard deviation to visualise the statistical noise in the correlation estimator in the form of an error bar in the correlation bar plots. The full width at half height (FWHH, also called "half-width") of a Gaussian distribution with a standard deviation of σ would be roughly 2.355×σ. So with σ=0.0761, we would have a FWHH of 0.18. Roughly, this resulting FWHH number is consistent with the width of our example bootstrap histogram at its half height. Our interpretation of this bootstrap histogram is that the distance of the point estimate to zero is significantly larger than the statistical noise in the correlation estimator, visualised by the width of the histogram around the mean value: The peak has a distance of μ/σ=4.9 or μ/FWHH=2 from zero.

We conclude that this analysis strongly supports our hypothesis that NIP and investor demand are negatively correlated. This confirms the assumption of an economical strategy for the NIP pricing.

Apparently, the dataset does hardly contain orderbooks with very low demand and very small NIP, which is not surprising, as the funding team would have increased the NIP to improve demand already before the final NIP was set. In the opposite scenario of an unexpectedly strong demand, the funding officers might have even increased the issue size beyond decreasing the NIP. Hence, it is not surprising that the dataset does not include orderbooks with very high demand and very high NIP.

**Figure 7.**
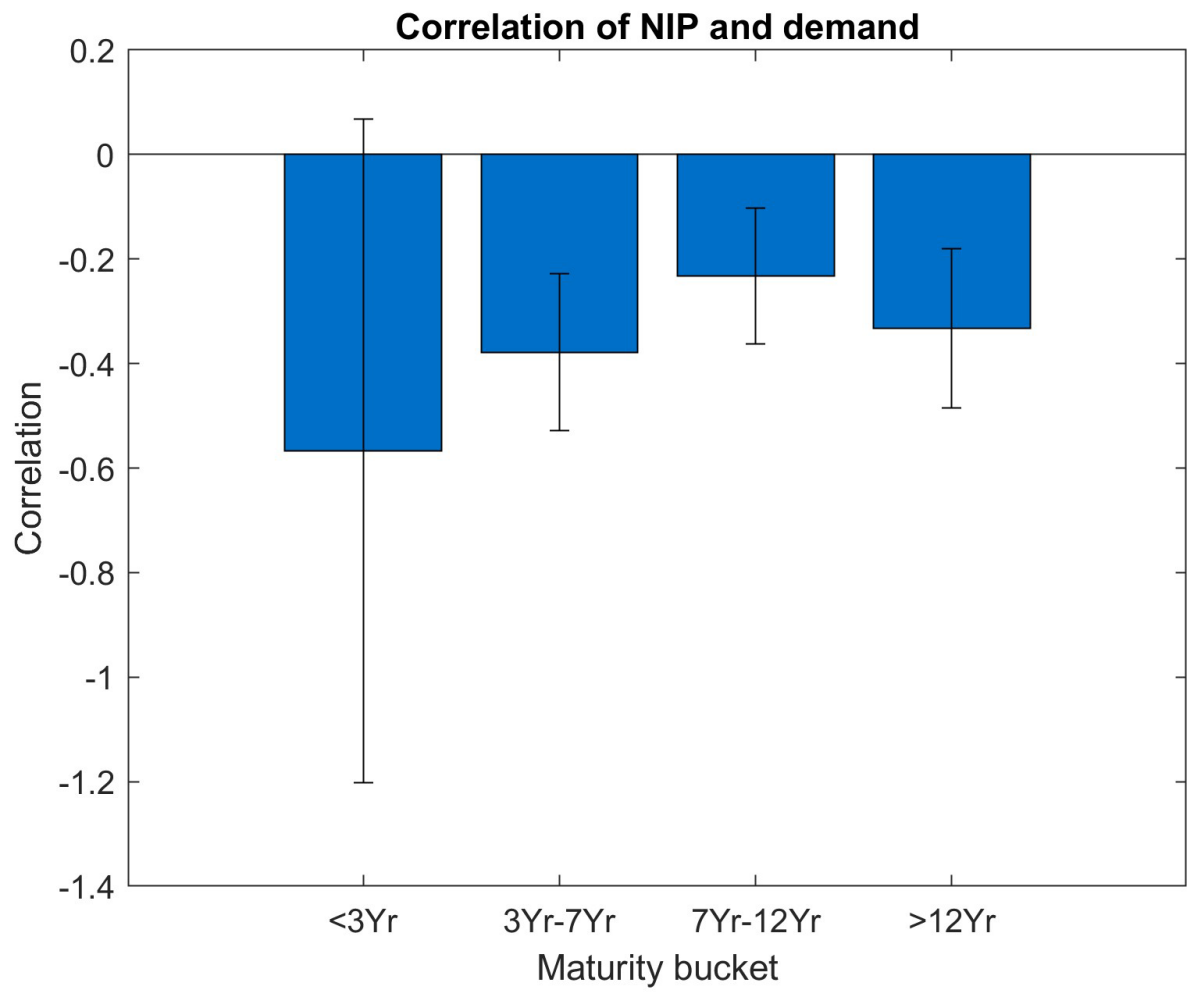
Correlation of NIP and demand per maturity bucket. EFSF & ESM bonds primary market.

**Figure 8.**
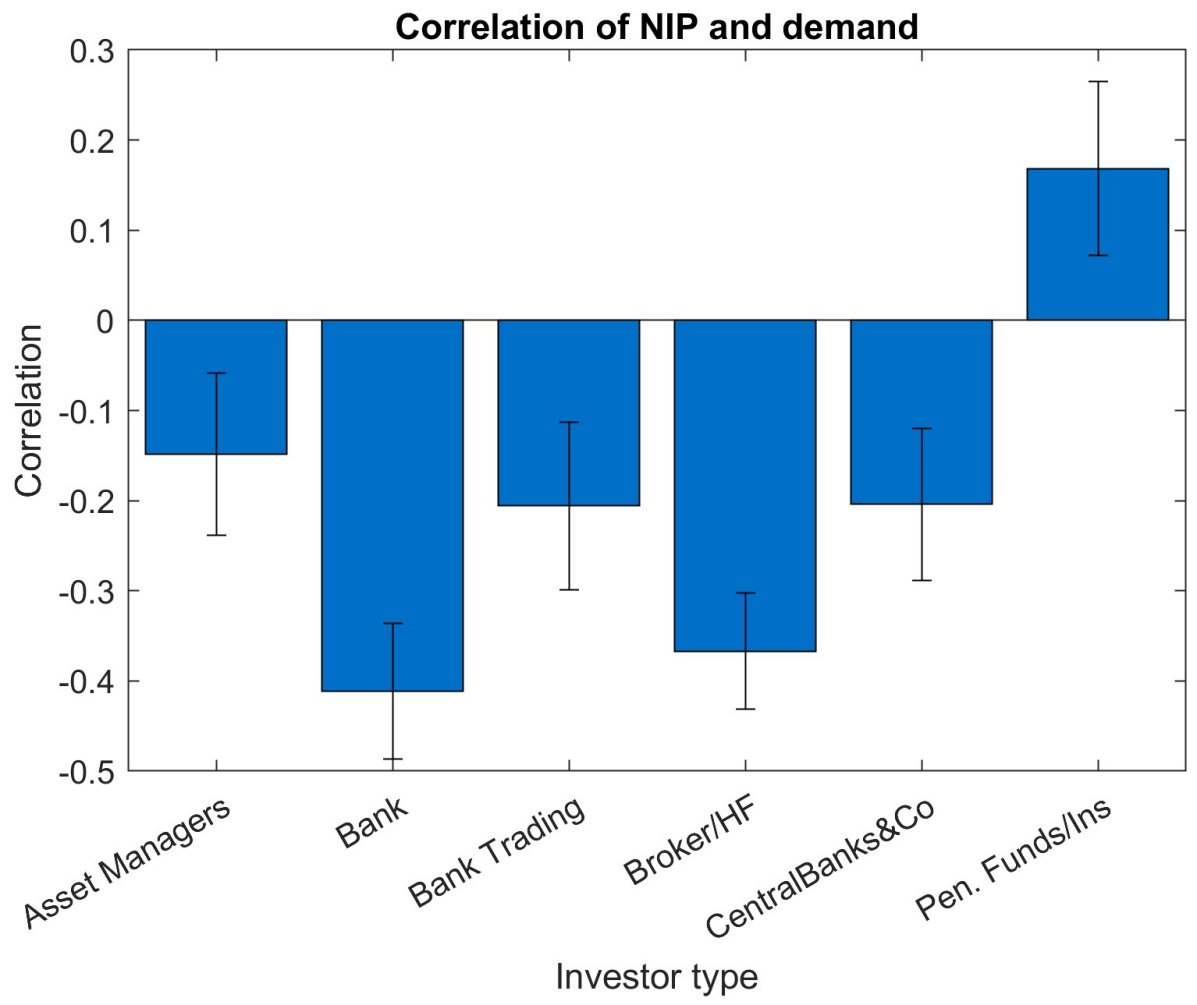
Correlation of NIP and demand for different investor types. EFSF & ESM bonds primary market.

In Figure 7 and Figure 8, we have a more granular look at the correlation between NIP and investor demand, at the same time we translate the bootstrap results to a correlation bar graph with additional error bars, the width of the error bars reflects the standard deviations. In order for the point estimate to be statistically significant beyond the noise, the error bar around the point estimate should show a substantial distance to the zero level.

Figure 7 shows the correlations between NIP and the total orderbook demand sorted into maturity buckets. Across all maturity buckets, the correlation estimate (i.e. the y axis value of the filled blue bars) is negative, but the standard deviation of the bootstrap (i.e. the length of the vertical error bar) is substantial. For maturities below three years, the correlations are not significant as the error bar also includes the zero-correlation value on the y axis. This can be explained by the smaller number of issuances in this bucket.

A similar bar chart with investor-type buckets instead of maturity buckets ( Figure 8) shows negative correlations for all investor types except for insurances and pension funds. The highest significance of the correlations can be observed for the two investor types "Banks" and "Brokers/Hedge Funds", as those show the most negative correlations and the smallest relative standard deviations. Due to their shorter holding periods and more tactical behaviour, those two investor types are not considered as strategic investors. Typically, the NIP would not be used to attract more demand specifically from those two investor groups. However, the stronger and more significant negative correlations of these two investor groups suggest that at a situation of high demand from these investors, the new issue premia are lowered and therefore not used as a tool to generate additional demand. Investors' "Pension Fund/Insurance" bucket shows significant positive correlations between order amount and NIP.

## Are there indications of orderbook inflation?

The following analysis investigates what the main drivers for large orderbooks are. Clearly, large orderbooks result from either a collection of large orders, a large number of orders, or a combination of both. While the number of orders from many different investors clearly stems from a broad interest among the investor base, larger orders could be stemming from either genuine demand or else by strategic considerations: in expectation of a large orderbook and a small allocation share, investors might increase their orders which again would further increase the overall orderbook size. Such a dynamic would lead to extremely larger orderbooks consisting of unusually large orders.

In this analysis, we look for indications that may indicate that investors tactically deviate from their real demand. Macroeconomic or market specific factors that change genuine or “real” demand of investors are not considered here. Hence we look for patterns that are unlikely to be triggered by economic factors, such as extremely large behavioural changes out of proportion with macroeconomic changes, or different behaviour of investors in the same economic environment.

**Figure 9.**
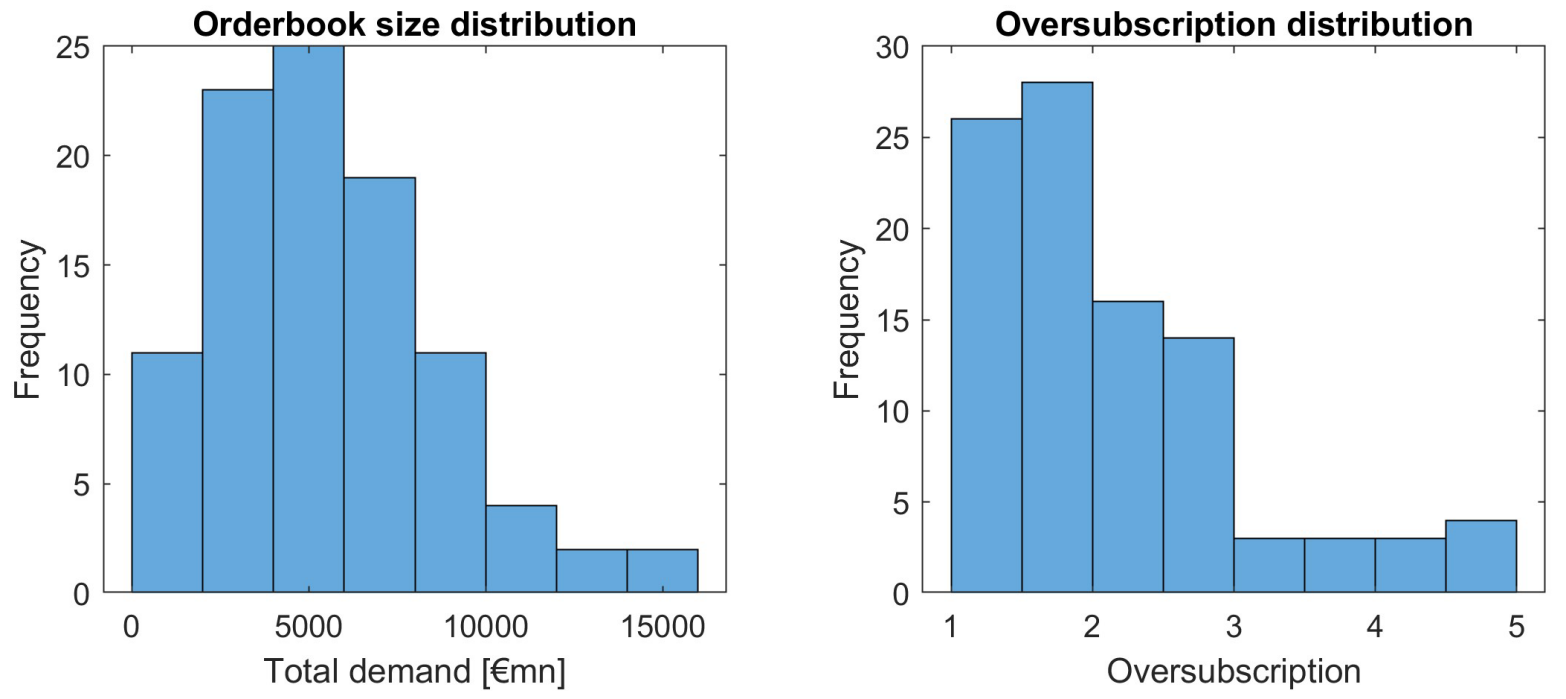
Distribution of orderbook and oversubscription as a function of total demand and oversubscription. EFSF & ESM bonds primary market.

Let us first have a look at the distribution of total demand across all issuances in absolute terms and relative to the issuance volume, see Figure 9.

We observe quite a variation in orderbook volume and relative terms (oversubscription), yet larger oversubscriptions are happening with decreasing frequency. Interesting is the "tail" with oversubscriptions larger than 4, which seems to be outside a natural distribution of orderbook sizes.

**Figure 10.**
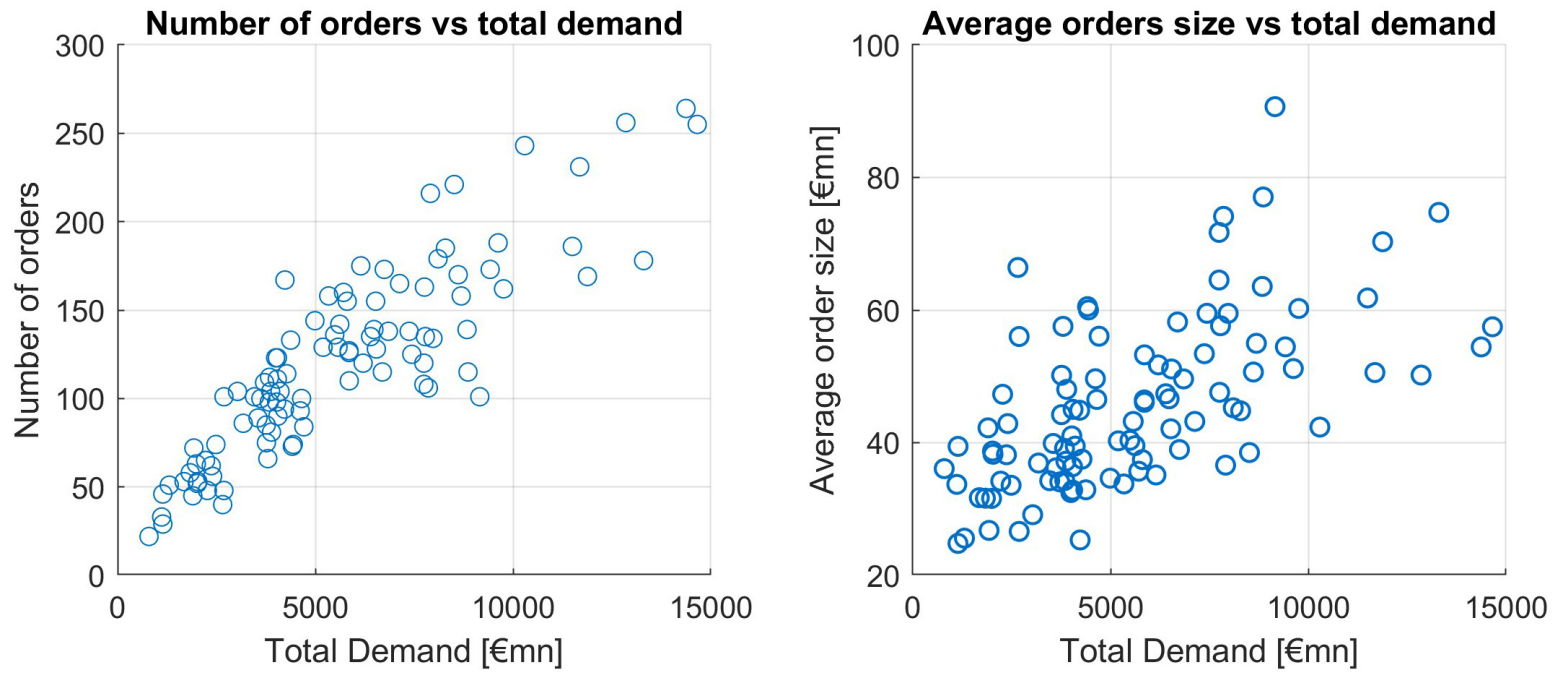
Number of orders and average order size as a function of total demand. EFSF & ESM bonds primary market.

Let us now look at the size and number of orders in relation to the orderbook size. Figure 10 shows that in ESM and EFSF issuances, large orderbooks rather stem from an increased number of orders and a moderate increase in the average order size.

A simple linear regression (without intercept) shows a significant impact of the number of orders on the orderbook size (total demand) (p-value < 0.01%), and this model has an R-squared of 76.2%. Hence the correlation between number of orders and total demand is 87.3%. Another way to show how much of the large orderbooks is explained by the order number is as follows, see Table 3.

**Table 3.** Averages of small and large order books. EFSF & ESM bonds primary market.

	Avg orderbook (bn)	Avg order (mn)	Avg order number
Books ≤ 5bn	3.16	39.64	82
Books ≥10bn	12.57	57.74	223

Assuming the average order size from small orderbooks of 39.64mn, the average number of orders of large orderbooks (223) would lead to an orderbooksize of 8.80bn, which is 70.26% of the average orderbook size of large orderbooks. In other words, more than 70% of a large orderbook can be explained by the larger number of orders in comparison to smaller orderbooks.

This strongly supports our hypothesis that the number of orders is a dominant driver of the orderbook size.

The average order size correlates with total demand on a much weaker level, at a point estimate of 0.6109. The scatterplot of average order size versus total demand is upward sloping, but this effect is weaker than the one of the number of orders, as seen in Figure 10.

**Figure 11.**
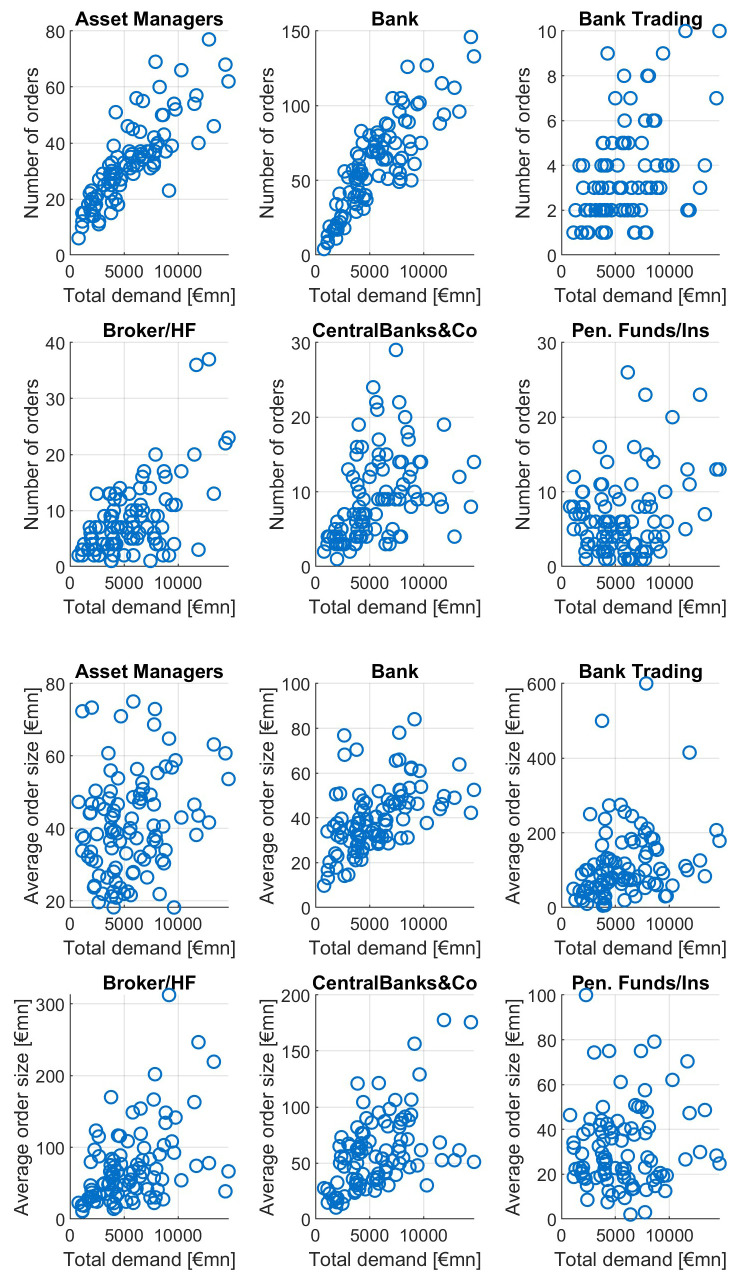
Number of orders and average order size as a function of total orderbook demand for different investor types. EFSF & ESM bonds primary market.

This pattern is not equally strong among different investor types, as can be seen in Figure 11. For increasing orderbook size we see increasing numbers of investors from asset managers, banks, brokers and CBs, with particular high correlations for asset managers and banks. The average order size tends to grow with growing total demand mainly for banks and asset managers, to a lesser extent for brokers/HF and central banks. Neither number of orders nor average orderbook size of pension funds is showing a clear dependence pattern relative to total demand.

As already noted at the beginning of this section, a higher average order size can be both caused by an effect of a market environment leading to an increase of demand from many investors and by a tactical behaviour of investors in expectation of a large orderbook. In the latter case, such a book would be called an "inflated orderbook" if it would be the result of tactical (inflationary) behaviour of many investors. Since we do not know whether the order of an investor is tactically deviating from its real demand, we cannot directly identify inflated orders. Instead, we are looking at exceptionally large orders, or "oversized" orders, defined on a granular investor level. If such oversized orders jointly occur in large orderbooks, this could be an indication of an inflated orderbook: investors would expect that other investors would increase their order in expectation of a large orderbook and hence increase their order.

**Figure 12.**
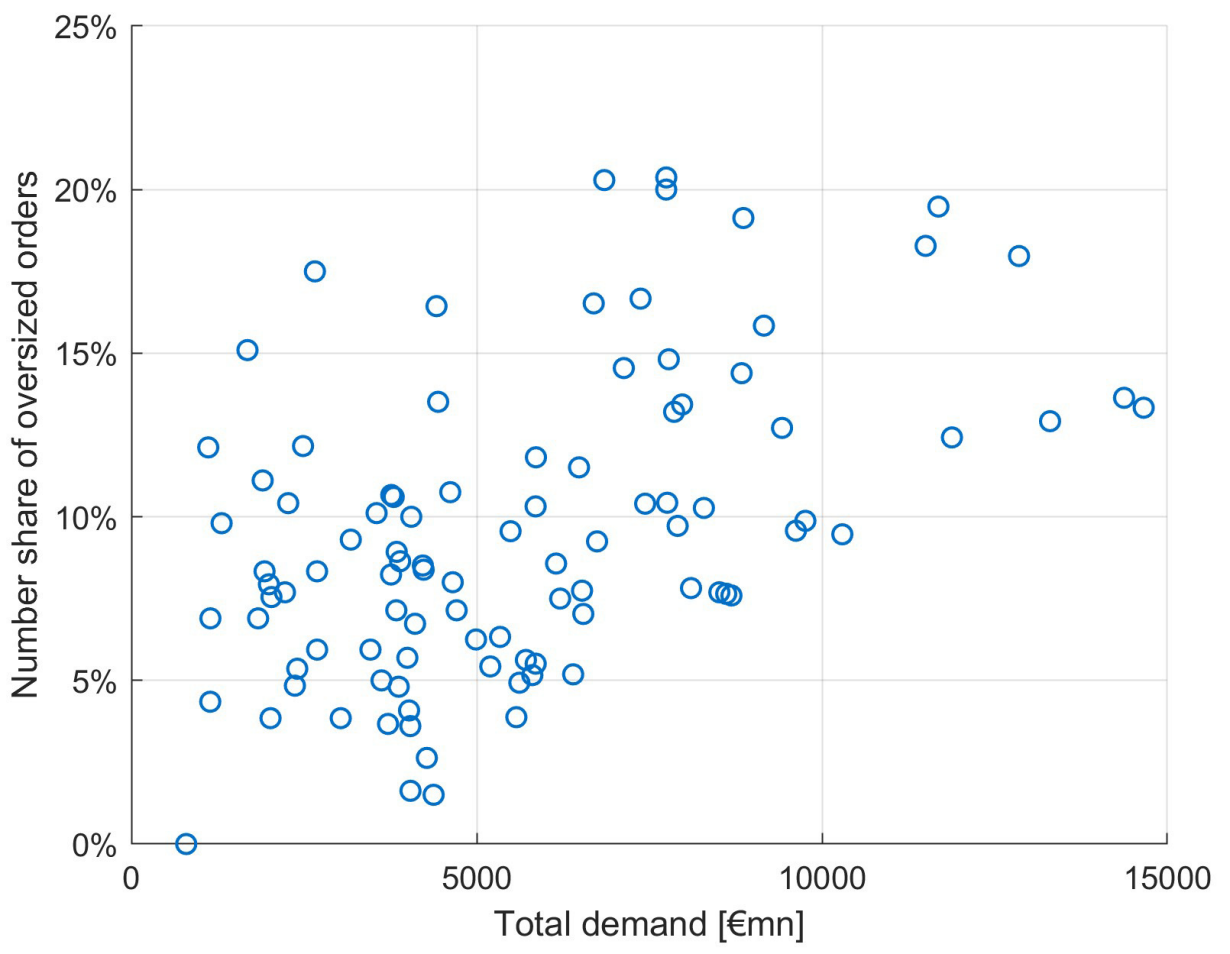
Number share of oversized orders as a function of total orderbook demand. EFSF & ESM bonds primary market.

Assuming that each investor has a certain level of regular investment, we look at large deviations from the average order volume of this specific investor. Obviously, we need to define a threshold regarding the order volume relative to a "regular" order volume. In this study we define an order as being *oversized* when it is larger than three times the median order of this investor. This is a heuristic approach, and other thresholds deliver similar results. We can characterise an inflated orderbook by the simultaneous occurrence of many oversized orders. Hence, such an inflated orderbook would be particularly large. Indeed, we see an increased share of oversized orders for larger orderbooks, yet they also appear in smaller orderbook size issuances as seen in Figure 12. Hence not all oversized orders can be viewed as inflated orders.

**Figure 13.**
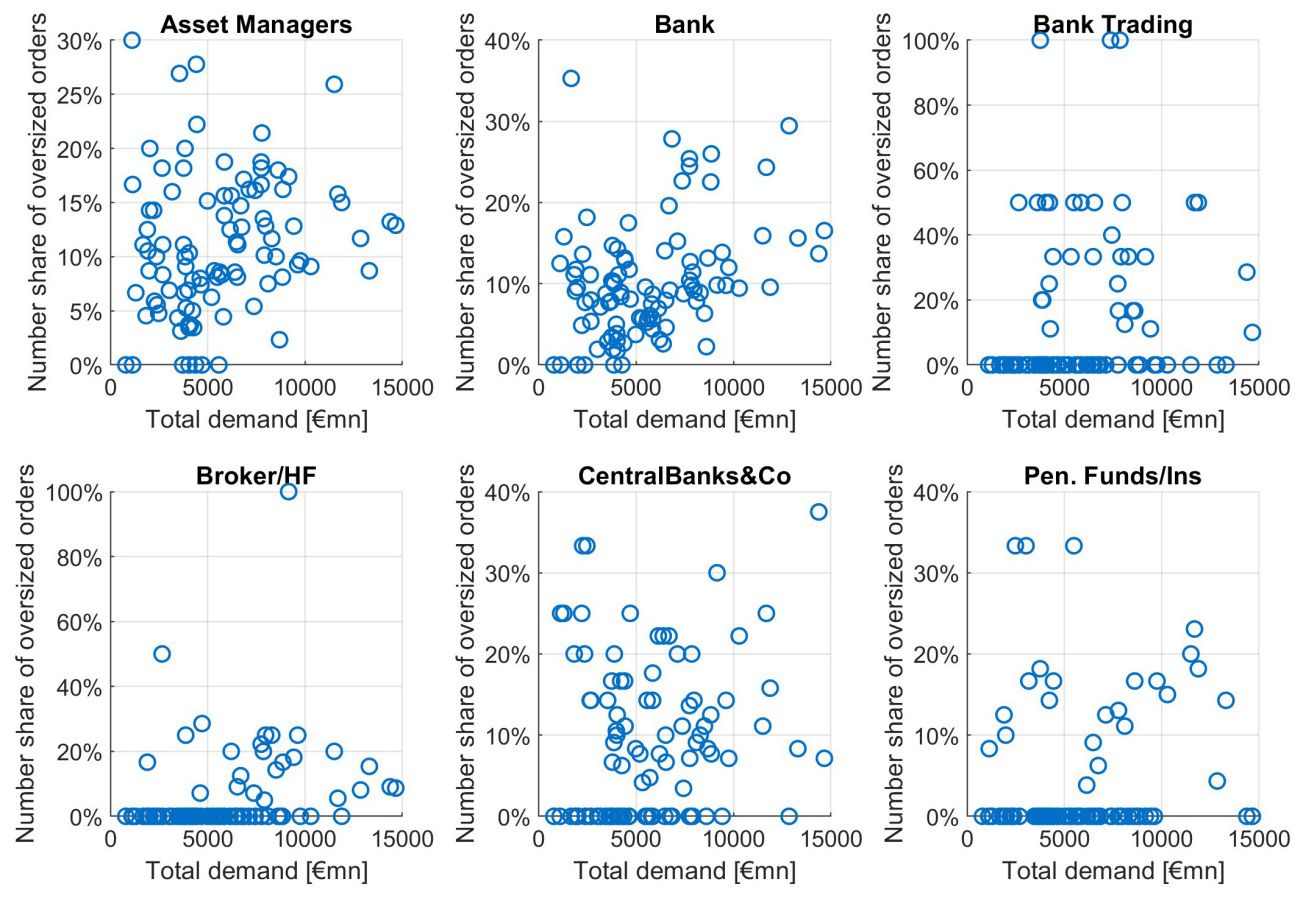
Number share of oversized orders as a function of total orderbook demand for different investor types. EFSF & ESM bonds primary market.

Looking at the different investor types ( Figure 13), we see many oversized orders in large orderbooks only for banks and asset managers to a lesser extent. Bank trading, broker/hedge funds, central banks and pension funds/insurances do not show the characteristic pattern of an increasing share of inflated orders as a function of total demand.

We evaluate this pattern of oversized orders in large orderbooks as follows: we look at the share of oversized orders among the different investor types. Since central banks and other public entities do not inflate their orders since they can expect a high allocation share (of usually more than 90%), we regard their order size distribution as "normal". Notably, we count 9% oversized orders for central banks and other public entities. In contrast, banks have 10.6% and asset managers even have 11% oversized orders. We deduct from this that banks and asset managers only have 1.6%–2% more oversized orders, not enough to assume inflationary behaviour behind it. We conclude that orderbook inflation has not been a significant feature in the EFSF and ESM syndicated transactions between January 2014 and September 2020.

## Conclusions and outlook

This study focuses on stylised facts of investor demand dynamics in syndicated bond transactions, namely regarding the main constituents segmenting investor demand, the pricing dynamics concerning investor demand, and a possible explanation of the apparently large orderbook size volatility.

The tranche of the issuance (benchmark or tap) and the tenor are main driving factors of investor demand. A benchmark issuance has on average a significantly larger orderbook than a tap. Further, we observe that the pattern of lower demand for larger tenors is significant for benchmark issuances only, while rather the opposite is true for tapped issuances, yet not significantly. Overall, across benchmark and tapped issuances, the different investor demand in the different tenor buckets is well reflected by the choice of the notional, implying an almost constant oversubscription across tenor buckets. This indicates that market demand for higher tenors overall matched the corresponding ESM/EFSF supply well.

We observe that the new issue premium (NIP) has been used sparsely in ESM/EFSF transactions in the sense that it had a significant tendency to be higher in market circumstances that were showing less investor demand and lower in market circumstances that were showing higher investor demand. This indicates an economical issuance practice where the pricing was well considering investor demand at different price levels.

Lastly, we do not find indications for a significant orderbook inflation in ESM/EFSF transactions in the analysed period. We base our analysis on the definition of "oversized orders" as those deviating substantially from the median order of each investor. We observe an increasing number share of oversized orders as a function of total demand and find a larger share of oversized orders from banks and asset managers, but not as a significant feature on the aggregated orderbook of all investors. Instead, we show that the number of orders is the main (highly significant) driver of investor demand, with an explanatory power of over 76%. This indicates that orders in syndicated orderbooks are a good measure of investor demand in the primary market.

Future research is progressing along two primary avenues. Firstly, amidst growing ESG awareness, we are exploring investor demand for green bonds and how it differs systematically from that of conventional bonds, ( Hillebrand & Thier, 2024). Moreover, we are exploring to include environmental cost beyond the carbon footprint into the pricing of financial instruments and into portfolio allocation decision-making ( Posth *et al.*, 2024). Secondly, we are interested to extend our analysis to the secondary bond market, where investors engage in trading bonds among themselves. We also would like to encourage other public issuers to collaborate. A larger consolidated dataset could enable multivariate analyses of the drivers of supply and demand across different public issuers and overcome an important limitation of this study.

## Ethics and consent

Ethical approval and consent were not required.

## Data Availability

The data underlying the results cannot be shared due to confidentiality agreements of the European Stability Facility (EFSF) and the European Stability Mechanism (ESM). The Methods section contains detailed information to allow replication of the study with a similar dataset from another bond issuer. Any queries about the methodology should be directed to the corresponding author.

## Footnotes

^2^ “Benchmark bonds” are reference bond from another issuer (in this case, from Germany). These are to be distinguished from the “benchmark issuances” of the same issuer (in this case, EFSF/ESM) in contrast to “tapped issuances”, i.e. an increased size of the same issuance, as explained in the next section.

^3^ See the definition of the CME treasure note and treasury bond futures: https://www.cmegroup.com/trading/interest-rates/us-treasury.html

^4^ See the definition of the Eurex note and bond futures: https://www.eurex.com/ex-en/markets/int/fix/government-bonds
