# Supplementary material for: Investor demand in syndicated EFSF/ESM bond issuances
Source: Open Res Eur. 2024 Jul 16;3:96. Originally published 2023 Jun 14. [Version 2] doi: 10.12688/openreseurope.15961.2 (PMC10446073; doi:10.12688/openreseurope.15961.2)
Supplement: Peer review history

# Reviewer report: Reviewer response for version 2

**DOI:** 10.21956/openreseurope.19562.r42216

**Published:** 2024-07-30

**Authors:** Gábor Sztanó

**Affiliations:** 1 Corvinus University of Budapest, Budapest, Hungary

I am pleased to read that the Authors addressed my concerns and I am glad to accept the current version of the manuscript.

If applicable, is the statistical analysis and its interpretation appropriate?

Yes

Is the study design appropriate and is the work technically sound?

Yes

Is the work clearly and accurately presented and does it engage with the current literature?

Yes

Are the conclusions drawn adequately supported by the results?

Yes

Are sufficient details of methods and analysis provided to allow replication by others?

Not applicable

Are all the source data and materials underlying the results available?

No source data required

Reviewer Expertise:

International finance

I confirm that I have read this submission and believe that I have an appropriate level of expertise to confirm that it is of an acceptable scientific standard.

# Reviewer report: Reviewer response for version 2

**DOI:** 10.21956/openreseurope.19562.r42218

**Published:** 2024-07-24

**Authors:** Patrick McColgan

**Affiliations:** 1 Department of Accounting and Finance, University of Strathclyde, Glasgow, UK

The authors have addressed the comments raised by all three reviewers to a reasonable degree.  I accept the response around revisions to the data description and justification for the focus on multivariate analysis in the work.  

 I would recommend further work to explain sample construction.  I think this element needs to significantly more descriptive of filtering and steps to construct the sample presented and more explanation of the proprietary database used in the work.

If applicable, is the statistical analysis and its interpretation appropriate?

Partly

Is the study design appropriate and is the work technically sound?

Yes

Is the work clearly and accurately presented and does it engage with the current literature?

Yes

Are the conclusions drawn adequately supported by the results?

Yes

Are sufficient details of methods and analysis provided to allow replication by others?

Partly

Are all the source data and materials underlying the results available?

Partly

Reviewer Expertise:

Restructuring, Employee layoffs, Security issuance

I confirm that I have read this submission and believe that I have an appropriate level of expertise to confirm that it is of an acceptable scientific standard, however I have significant reservations, as outlined above.

# Reviewer report: Reviewer response for version 2

**DOI:** 10.21956/openreseurope.19562.r42217

**Published:** 2024-07-24

**Authors:** Athanasios Tsagkanos

**Affiliations:** 1 Department of Business Administration, University of Patras, Patras, Greece

The authors follow my instructions and now the paper is ready for indexing.

If applicable, is the statistical analysis and its interpretation appropriate?

Yes

Is the study design appropriate and is the work technically sound?

Yes

Is the work clearly and accurately presented and does it engage with the current literature?

Yes

Are the conclusions drawn adequately supported by the results?

Yes

Are sufficient details of methods and analysis provided to allow replication by others?

Yes

Are all the source data and materials underlying the results available?

Yes

Reviewer Expertise:

Financial management, econometrics, portfolio analysis, applied economics.

I confirm that I have read this submission and believe that I have an appropriate level of expertise to confirm that it is of an acceptable scientific standard.

# Reviewer report: Reviewer response for version 1

**DOI:** 10.21956/openreseurope.17235.r33971

**Published:** 2023-08-10

**Authors:** Gábor Sztanó

**Affiliations:** 1 Corvinus University of Budapest, Budapest, Hungary

The article discusses stylized facts related to ESM/EFSF bond issuances. The applied methodology is quite simple but in my view the paper reaches its goal in terms of sharing basic statistical features of the bond issuers that has had a great importance in dealing with sovereign funding crises in the 2010s.  

 I would point out some minor and a couple of major recommendations in connection with the manuscript. 

**Major: **

1. Inflated order book could be a result of 'market-testing', when participants on the auctions submit more bids with different pricings. They usually do this to ensure they will reach the amount of bonds they intend but they are unsure of other participants' bids. Honestly, I am not sure how EFSF/ESM auctions work, but even if it is not an issue (because of the feature of  the auctions) authors should note this. 
2. I would  recommend further analysis on the story behind Figure 1. It looks like yield premium over German bund increased around 2016-7 and it may be interesting what determined this spread. But it is rather a suggestion for further research than an actual criticism. 

**Minor: **

1. I have found footnote 2 about benchmark and benchmark issuance but I found still confusing in the text. For the premia over German bonds, Bund-spread or spread over Bund benchmark would be clearer. 
2. It was not very meaningful to test the null hypothesis of the smaller estimate being actually smaller than the bigger one. Equality of these would make more sense from a perspective of formal testing, though I believe that it would not really change the outcome. 
3. I think Table 1 was misplaced, it would be better in the following section. 
4. "Our main finding is that demand is larger for smaller tenors, except for the short maturities (<3Yr) which is the tenor bucket with the fewest issuances, see the left hand side panel of Figure 3. " -- The number of issuances is in Table 1, probably a reference to that table would be an idea. Figure 3 says nothing about the issuances (so rephrasing could also help). 

All in all, considering the overall quality of the manuscript, I approve the paper.

If applicable, is the statistical analysis and its interpretation appropriate?

Yes

Is the study design appropriate and is the work technically sound?

Yes

Is the work clearly and accurately presented and does it engage with the current literature?

Yes

Are the conclusions drawn adequately supported by the results?

Yes

Are sufficient details of methods and analysis provided to allow replication by others?

Not applicable

Are all the source data and materials underlying the results available?

No source data required

Reviewer Expertise:

International finance

I confirm that I have read this submission and believe that I have an appropriate level of expertise to confirm that it is of an acceptable scientific standard.

# Response

**Published:** 2024-06-26

**Authors:** Peter Schwendner

**Affiliations:** Institute of Wealth and Asset Management, Zurich University of Applied Sciences, Winterthur, ZH, Switzerland

Dear Gábor,   

 Thank you very much for your helpful review! We have addressed your points as follows:

 Major

1. **Mechanism of orderbook inflation**: we have further explained it at the end of the introduction.
2. **Background of** **Figure 1**: the premium increase in 2016 is mainly due to a liquidity decrease with start of the quantitative easing of the ECB.

Minor

1. We have clarified the footnote and the text accordingly.
2. Indeed - the chart should be in the next section. 
3. The hypotheses have been chosen this way on purpose. E.g. we do not want to show that benchmark and tap issuances have different demand, but that benchmark issuances have more demand than taps.
4. We have added a reference to Table 1 accordingly.

Kind regards Peter

# Reviewer report: Reviewer response for version 1

**DOI:** 10.21956/openreseurope.17235.r33144

**Published:** 2023-07-27

**Authors:** Athanasios Tsagkanos

**Affiliations:** 1 Department of Business Administration, University of Patras, Patras, Greece

I appreciate the chance to serve as a reviewer on this paper. The paper is well written and suits the scope of the issue. I urge the author(s) to consider the following comments and improve the paper accordingly.

1. At first, I think the authors should make clearer the economic meaning of their research starting with an economic motivation.
2. I think the introduction is too long which somehow makes the reading difficult. Therefore, it is better for it to be divided into two sections (introduction and literature review).
3. I would like the authors to make a prediction about investor behavior in the near future in similar financial conditions where green bond issuance has been overwhelming.
4. It would also be useful for the audience and future researchers if a guide for the future research is provided: how could this research be used concretely to open new pathways? Is it possible to provide some examples and possible directions for future research? 

If applicable, is the statistical analysis and its interpretation appropriate?

Yes

Is the study design appropriate and is the work technically sound?

Yes

Is the work clearly and accurately presented and does it engage with the current literature?

Yes

Are the conclusions drawn adequately supported by the results?

Yes

Are sufficient details of methods and analysis provided to allow replication by others?

Yes

Are all the source data and materials underlying the results available?

Yes

Reviewer Expertise:

Financial management, econometrics, portfolio analysis, applied economics.

I confirm that I have read this submission and believe that I have an appropriate level of expertise to confirm that it is of an acceptable scientific standard, however I have significant reservations, as outlined above.

# Response

**Published:** 2024-06-26

**Authors:** Peter Schwendner

**Affiliations:** Institute of Wealth and Asset Management, Zurich University of Applied Sciences, Winterthur, ZH, Switzerland

Dear Athanasios,

 Thank you very much for your helpful review! We have addressed your points as follows:

1. **Economic motivation**: we explained the economic motivation in the introduction.
2. **Long introduction**: we split the introduction into two chapters, according to your suggestion.
3. **Green Bonds**: the effect on green bonds is a topic that we are working on in a separate paper. We have addressed this point in the Outlook.
4. **Further Research**: Another path of further research is a look into secondary market data. We have added this point to the Conclusions.

Kind regards Peter

# Reviewer report: Reviewer response for version 1

**DOI:** 10.21956/openreseurope.17235.r33146

**Published:** 2023-07-24

**Authors:** Patrick McColgan

**Affiliations:** 1 Department of Accounting and Finance, University of Strathclyde, Glasgow, UK

The article examines drivers of orderbook demand, pricing factors, and measures of volatility in demand for a sample of bonds issued under the EFSF/ESM scheme between 2014 and 2020. The study uses a proprietary data set of bond issue, investor demand and individual investor orders to examine these key research questions. The analysis is generally descriptive and largely focused on a univariate type setting where the researchers examine a single factor as predictors of the main research questions examined in the study. The main findings are interesting and largely consistent with prior literature but nonetheless the unique data set allows the authors to address some novel aspects in these research questions that are otherwise difficult to explore.

 My main concerns are around the explanation of relevant theory, the largely univariate nature of the analysis, and the ability to explain and replicate the findings as described in the paper.

1. Univariate nature of analysis – much of the empirical analysis is descriptive and focuses on the role of a single explanatory variable. For example, when analysing investor demand (oversubscription) the study focuses only on tenor of the bonds issued. There is a notional split in the data set between benchmark bonds and taps, but otherwise there are a range of additional firm level factors (issuing firm size, bond issue size, current leverage, etc) that might also predict demand and be correlated with the tenor of the bond. The authors may argue that some of these metrics are picked up in the oversubscription variable of investor demand divided by bond issue size, but even here it would still be relevant to control for whether these variables have a residual effect on demand. Similar comments apply for analysis of bond pricing/new issue premium, and volatility of investor demand. Many of the academic studies cited in this paper can give directions in relevant controls in a multivariate setting.
2. Theoretical motivation – the descriptive nature of the analysis is appropriate and common in practitioner focused work, but I would also expect to see some further discussion in the introduction to the paper and through the motivation for tests on what a priori expectations are in the study. There is some work in this area around investor demand, but I found this weak in the remainder of the paper and also in the motivation for empirical tests. As an example, when tenor and benchmark/taps status is used to segment oversubscription I don’t think the paper does enough to explain what the correct tenor buckets should be (two different splits are used in the paper) and why we should expect differences in the two main issue types.
3. Data description – while appreciating that the data is described as proprietary, I would still expect to see a lot more work around sample construction, data filtering, treatment of different tranches of the same bond (if applicable), and general description of the data available and its source to aid replication.  I found this aspect of the paper unnecessarily weak. The authors should look at published work that uses proprietary from government or investment bank data sets on bond issuance for good models in explaining data without giving away unpermitted information on the proprietary nature of the data set.

If applicable, is the statistical analysis and its interpretation appropriate?

Partly

Is the study design appropriate and is the work technically sound?

Yes

Is the work clearly and accurately presented and does it engage with the current literature?

Yes

Are the conclusions drawn adequately supported by the results?

Yes

Are sufficient details of methods and analysis provided to allow replication by others?

Partly

Are all the source data and materials underlying the results available?

Partly

Reviewer Expertise:

Restructuring, Employee layoffs, Security issuance

I confirm that I have read this submission and believe that I have an appropriate level of expertise to confirm that it is of an acceptable scientific standard, however I have significant reservations, as outlined above.

# Response

**Published:** 2024-06-26

**Authors:** Peter Schwendner

**Affiliations:** Institute of Wealth and Asset Management, Zurich University of Applied Sciences, Winterthur, ZH, Switzerland

Dear Patrick,

 Thank you very much for your helpful review! We addressed your points as follows:

1. **Univariate nature of analysis**: we fully agree that a multivariate analysis would be the preferable approach, yet the data sample size needs to be larger to obtain significant results. Therefore, we seek to collaborate with other issuers to carry out a multivariate analysis on a joint data set. We have added a sentence in the Outlook that a multivariate analysis is envisaged on an extended data set.
2. **Theoretical motivation**: in the introduction, we have included both a priori expectations for our tests and also an explanation how the tenor buckets are selected.
3. **Data description**: the data description has been extended accordingly.

Kind regards

 Peter
